# Fermentation: A Boon for Production of Bioactive Compounds by Processing of Food Industries Wastes (By-Products)

**DOI:** 10.3390/molecules23102560

**Published:** 2018-10-08

**Authors:** Pardeep Kumar Sadh, Suresh Kumar, Prince Chawla, Joginder Singh Duhan

**Affiliations:** 1 Department of Biotechnology, Ch. Devi Lal University, Sirsa 125055, India; pardeep.sadh@gmail.com (P.K.S.); rohillasuresh@gmail.com (S.K.); 2 School of Bioengineering and Food Technology, Shoolini University, Solan 173229, Himachal Pradesh, India; princefoodtech@gmail.com

**Keywords:** bioactive compounds, solid state fermentation, sub-merged fermentation, liquid fermentation, food waste

## Abstract

A large number of by-products or wastes are produced worldwide through various food industries. These wastes cause a serious disposable problem with the environment. So, now a day’s different approaches are used for alternative use of these wastes because these by-products are an excellent source of various bioactive components such as polyphenols, flavonoids, caffeine, carotenoids, creatine, and polysaccharides etc. which are beneficial for human health. Furthermore, the composition of these wastes depends on the source or type of waste. Approximately half of the waste is lignocellulosic in nature produced from food processing industries. The dissimilar types of waste produced by food industries can be fortified by various processes. Fermentation is one of the oldest approaches and there are three types of fermentation processes that are carried out such as solid state, submerged and liquid fermentation used for product transformation into value added products through microorganisms. Selections of the fermentation process are product specific. Moreover, various studies were performed to obtain or fortified different bioactive compounds that are present in food industries by-products or wastes. Therefore, the current review article discussed various sources, composition and nutritive value (especially bioactive compounds) of these wastes and their management or augmentation of value-added products through fermentation.

## 1. Introduction

### 1.1. Bioactive Compounds

“Bioactive compounds” are made up of two words i.e., bioactive and compounds. Scientifically, it means various molecules that have some biological activity. Thus, a definition of bioactive compounds in plants is: secondary plant metabolites prompting pharmacological or toxicological effects in man and animals. Bioactive compounds are the phytochemicals that present naturally to lesser extents in plants as well as foodstuffs [1] and have the potential to amend metabolic processes for the promotion of better health. The typical bioactive compounds produced in plants are secondary metabolites not required for the circadian functioning of the plant. Bioactive compounds are extremely heterogeneous class of compounds includes plant growth factors, alkaloids, mycotoxins, food-grade pigments, antibiotics, flavonoids and phenolic acids etc. with dissimilar chemical structures (hydrophilic or lipophilic), specific to ubiquitous distribution in nature, significant amount present in foods and in human body, efficient against oxidative species and possess the potential biological action [2,3].

Bio accessibility and bioavailability do not necessarily depend on the abundance of bioactive compound in ingested food as well as the number of active metabolites in target tissues [4]. During investigating the role of each bioactive compound in human health their bioavailability is not always well defined [2]. Bioactive compounds are available in different concentrations in fruits, vegetables, and whole grains [5,6]. Several factors such as the source of food, molecular size, low lipid solubility, chemical interactions among the phytochemicals or biomolecules etc. may interfere with the bio-accessibility and bioavailability of bioactive compounds [7]. Bio-accessibility means releasing nutrient from the food matrix and turned into a chemical form enter in gut cells and making that nutrient bioavailable. The whole process comprises chewing, initial enzymatic digestion of the food in the mouth, mixing with acid and gastric juice, finally into the small intestine the food is assimilated and absorbed nutrients are rendered bio-accessible.

Different steps in food preparation involved cooking, chopping, or pureeing collaborate with mastication and enzymatic digestibility of food matrices [8]. Bioavailability is significantly affected by the release of bioactive compounds from plant matrix, solubility in the gastrointestinal fluid and their passage across intestinal epithelial cells, as well as the enzymatic and chemical reactions occurring within the GI tract [9]. Four steps are necessary for the effective absorption of bioactive compounds:

1. Releasing from the food matrix
2. Integration with bile-salt making micelles
3. Assimilation and absorption by epithelial cells, and finally
4. Incorporation into the chylomicrons with secretion into lymphatic system

Bioactive compounds absorption does not take place by simple diffusion processes as well as their inability to pass the lipid-rich outer membrane of the small intestine [10]. Various technologies like phytosome, nanocarriers, etc. have been developed not only to enhance bio-accessibility and bioavailability of bioactive compounds but also to protect active substances from oxidation or other degradation reactions in the gastrointestinal tract [11]. Another strategy for enhancing the bioavailability and bio-efficacy of bioactive compounds is altering their structure (prodrug strategy) to obtain a new structure having favorable kinetics and can also be transformed into the active form in an organism [11]. Furthermore, newer techniques are still developed in order to utilize bioactive compounds in the best possible way to get most of their health potential.

In recent years, excessive consideration towards bioactive compounds has been paid because of their aptitude for human health, such as decrease the rate of progressive and cardiovascular diseases like cancer, diabetes etc. [12,13]. They also exhibit anti-microbial, antioxidant, anti-mutagenic, anti-allergenic and anti-inflammatory properties, inhibition or induction of enzymes, inhibition of receptor activities as well as induction and inhibition of gene expression [14,15,16,17,18,19]. Table 1 shows various bioactive compounds present in different foodstuffs. Owing to favorable features for human health, researches have been intended to found that plants, vegetables, fruits, food industries and agro-industrial by products as low-priced sources for the bioactive compounds.

#### Synthesis and Purpose in Plants

Secondary metabolites are produced within the plants besides the primary biosynthetic and metabolic routes of compounds Primary metabolites like carbohydrates, amino acids, proteins, and plants aimed to promote their growth and development synthesize lipids. Besides this small amount of secondary metabolites are also produced within the plants. In the plant cells, secondary metabolites are considered as products of biosynthetic “side tracks” and are not required for the daily functioning of the plant. Phylogenetically, the secondary metabolites in plants seem to be randomly synthesized—but they are not useless junk. Several of them like flavonoids, phenolic acids etc. are found to perform different functions such as protecting a living plant against free radicals generated during photosynthesis. Likewise, terpenoids perform specific biological functions such as they may attract pollinators or seed dispersers, or inhibit competing plants. Similarly, alkaloids protect crop plants from herbivore and insect attacks (phytoalexins). Further, other secondary metabolites produced by common food and feed plants may function as cellular signaling molecules or they may perform different biological functions in the plants. However, the typical poisonous or medicinal plants sometimes contain a higher amount of more potential bioactive compounds than food and feed plants.

### 1.2. Types of Bioactive Compounds

**Figure 1:**
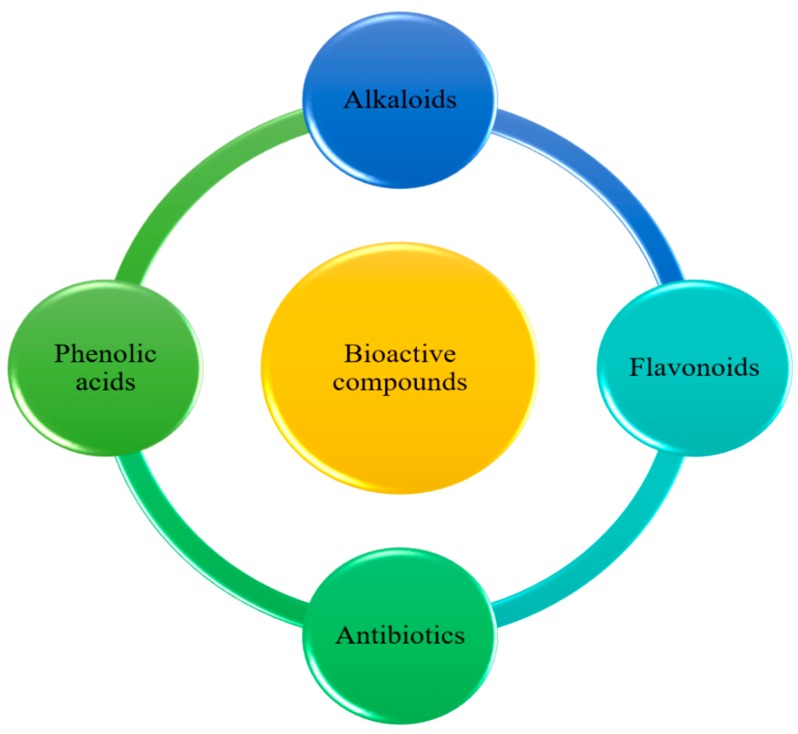
Bioactive compounds and their major types.

Bioactive compounds may influence physiological or cellular activities in the animals or humans after their consumption. Major classes of bioactive compounds (Figure 1) are described in the following section of this review.

#### 1.2.1. Flavonoids

**Figure 2:**
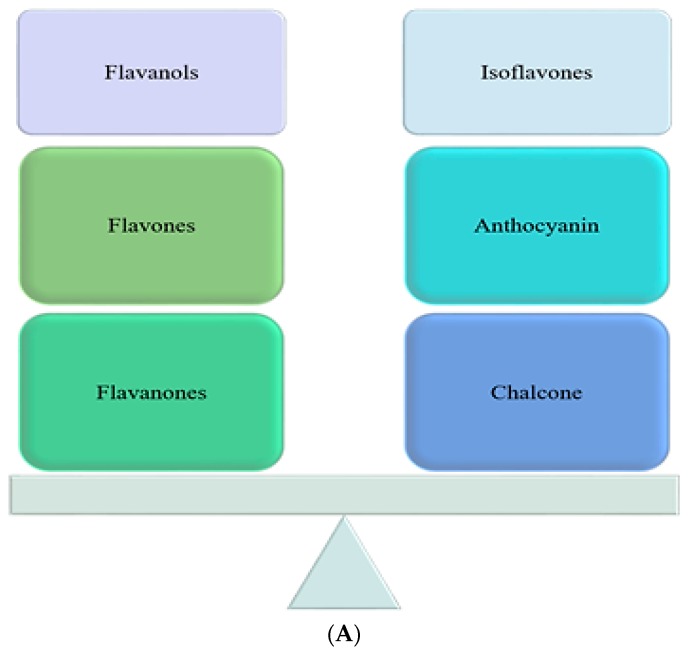

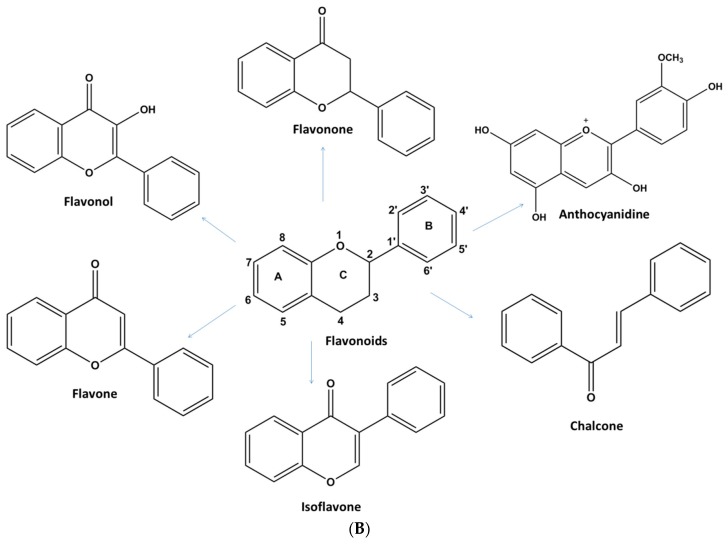
(**A**) Flavonoids and their types (**B**) Basic structure of common flavonoids.

Flavonoids found the prime group of naturally occurring phenolic compounds [50]. Flavonoids consist of constitutes of different types based on their chemical components e.g., flavonols, flavones, isoflavones, anthocyanin etc. (Figure 2A). These are low molecular weight compounds having a three-ring structure with several substitutions as shown in Figure 2B. Rutin, the first flavonoid was isolated from orange (genus: *Citrus*) in 1930; since then many flavonoids and their derivatives from different plants have been isolated and characterized with respect to their biochemical and biological activities [51].

##### Flavonols

Flavonols are ketone group containing flavonoids used for the biosynthesis of proanthocyanins. Kaempferol, quercetin, myricetin, and fisetin are most studied flavonols. Flavonols are abundant in lettuce, tomato, onions, apple, grape, berries, tea, and red wine etc. Consumption of flavonols reveals the wide range of health benefits including antioxidant property and reduced risk of vascular disease as well [52].

##### Flavones

Flavones are other important subgroups of flavonoids. They are abundantly found as glucosides in *Ginkgo biloba*, celery, parsley, mint, red peppers, citrus, and other fruits. Luteolin, apigenin, and tangeritin are the common flavones. Citrus fruits peels are rich in the polymethoxylated flavones such as tageretin, nobiletin, and sinensetin. They show different biological and pharmacological functions including antioxidant, antiallergic, anti-inflammatory, antiviral, anticancer and antitumor effects [53,54].

##### Flavonones

Flavonone is a small group of flavonoids specific for citrus fruits such as oranges, lemons, lime, and grapes, etc. Hesperitin, naringenin, and eriodictyol flavonones are responsible for the particularly bitter taste of the juice and peel of citrus fruits [55]. Flavonones have lots of health benefits because of their free-radical-scavenging properties (antioxidant) [56,57] as well as anti-inflammatory and cholesterol-lowering potential [58].

##### Isoflavones

Isoflavones are extensively studied the diverse subgroup of flavonoids. Their occurrence is limited in the plant kingdom, however significant amount found in soybeans and other leguminous plants in the form of glucosides. Further, a significant amount of aglycones has been reported in various fermented soy products [59,60]. Some isoflavonoids have also been reported in some microorganisms [51]. Isoflavones may use as precursors for the synthesis of phytoalexins during plant-microbe interactions. Isoflavonoids have tremendous potential to combat against different diseases. Isoflavones like genistein and daidzein are generally regarded as phytoestrogens due to their estrogenic activity in certain animal models [61,62].

##### Anthocyanins

Anthocyanins are an array of pigments accountable for colors in plants, flowers, and fruits. Cyanidin, delphinidin, malvidin, pelargonidin, and peonidin are the most studied anthocyanin. They are widely distributed in the outer layers of fruits such as cranberry, black currant, red grape, merlot, raspberry, strawberry, blueberry, bilberry, and blackberry. Anthocyanins are stable even when they exposed to different physiochemical conditions, which have a major influence on their structure. Anthocyanins have a broad range of pharmacological activities such as anti-inflammatory, antioxidant, antimicrobial, and anticarcinogenic activities [63]. Owing to health benefits of these compounds enable them to be used in the food industry in a variety of applications.

#### 1.2.2. Alkaloids

**Figure 3:**
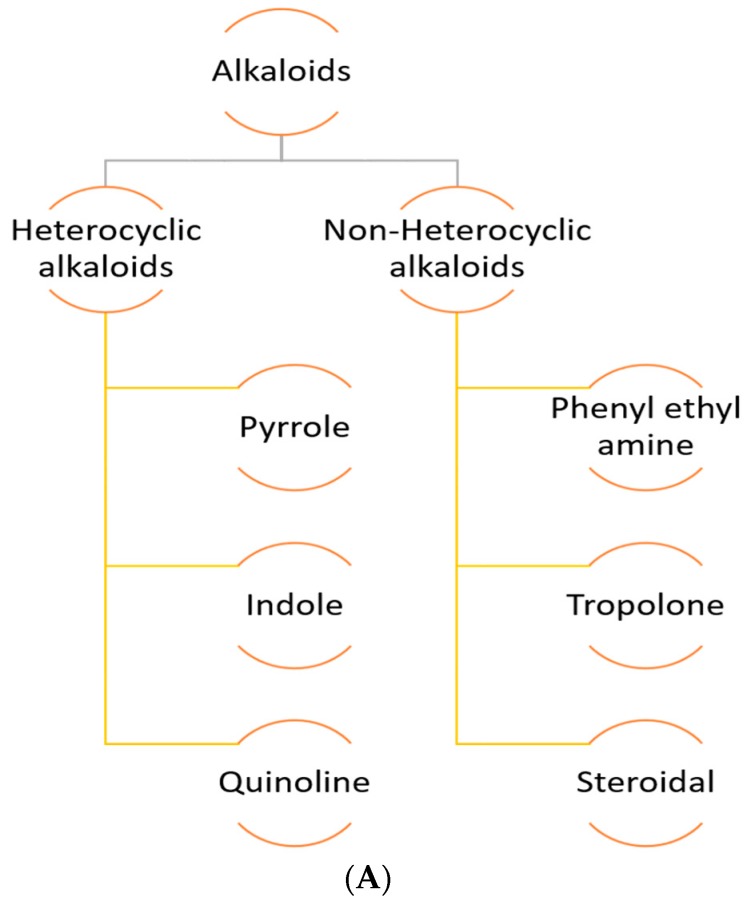

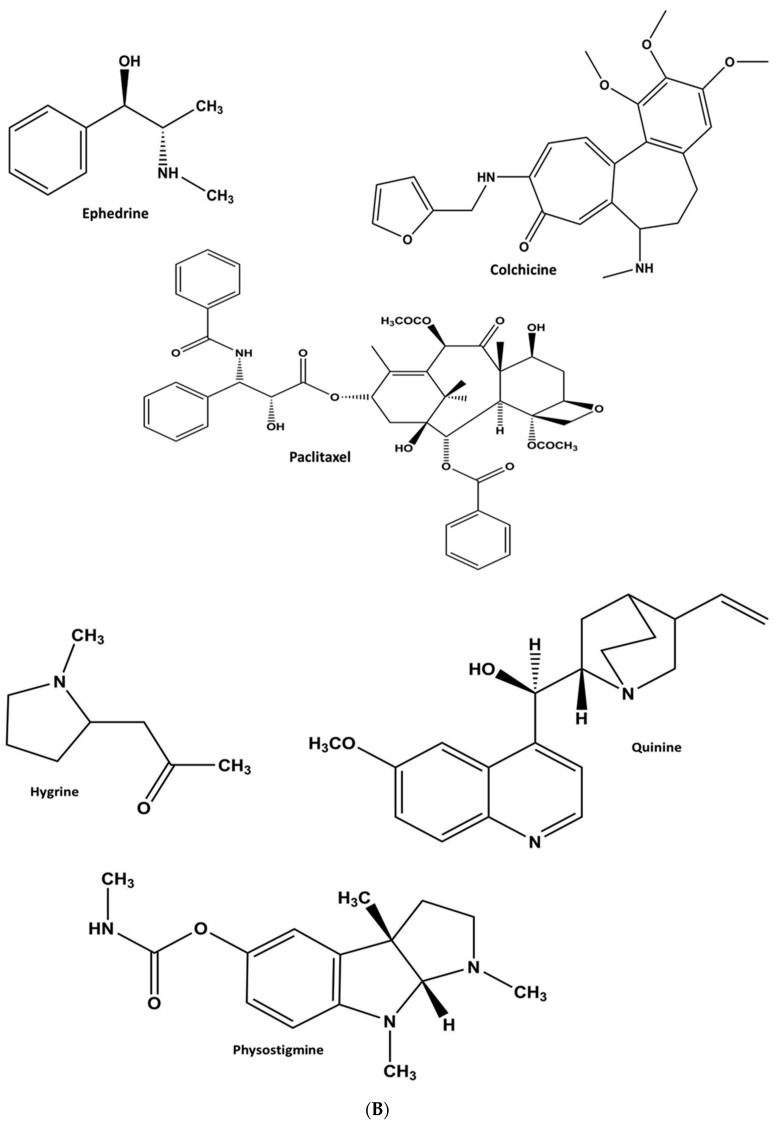
(**A**) Alkaloids and their types, Common structure of (**B**) Heterocyclic alkaloids and Non-heterocyclic alkaloids.

Alkaloids are a group of naturally occurring bioactive compounds composed of basic nitrogen atoms with bitter a taste. Figure 3A shows various types of alkaloids. Alkaloids are produced by a great variety of organisms including plants, animals and microorganisms to perform a specific physiological function. On the basis of whether the nitrogen is a part of ring or not, alkaloids are classified into type i.e., non-heterocyclic alkaloids or atypical alkaloids and heterocyclic alkaloids or typical alkaloids. The various groups have diverse pharmacological properties.

##### Heterocyclic Alkaloids

Structurally these have also nitrogen as a part of a cyclic ring system (Figure 3B). These are predominantly found in nature. Heterocyclic alkaloids are further subdivided into many groups based on the ring structure containing the nitrogen. Nevertheless, in this review some of the commonly used heterocyclic alkaloids are explained.

##### Pyrrole

Pyrrole is synthesizing by cyclization of 1,4-dicarbonyl compounds with an excess of ammonia or primary amines. It is a colorless volatile alkaloid and first identified by F. F. Runge [64]. Pyrroles and its derivatives are one of the most imperative classes of heterocyclic alkaloids. They exhibit widespread pharmacological and biological properties such as antibacterial [65], anti-inflammatory [66], antioxidant, antitumor [67], antifungal, cholesterol reducing [68] and immune suppressant activities, respectively [65].

Pyrrole itself is not a naturally occurring compound, but its derivatives are found in a variety of natural products as cofactors or coenzymes. Pyrrole is one of the main components of tobacco smoke and not as a constituent. Pyrrole is an important precursor to several drugs such as tolmetin, hygrine, stachydrine, atorvastatin, ketorolac, and sunitinib. Besides, vitamin B_12_, bilirubin, biliverdin, porphyrins, bacteriochlorins, porphyrinogens etc. are the compounds that contain pyrroles as an ingredient.

##### Quinoline

It is also a colorless hygroscopic alkaloid with a strong odor and was first noticed by F.F. Runge [64] from coal tar. Quinoline or benzo [*b*] pyridine is a nitrogen-containing heterocyclic aromatic compound. Fabaceae, Annonaceae, Moraceae, Rutaceae and Solanaceae are the excellent source of quinoline but Gymnosperms and Pteridophytes such as ferns and monocots contain this in very small amount. Most of the quinoline obtained from plants; however, some alkaloids have also been isolated from algae, fungi, insects, marine and land animals as well. Quinine, quinidine, cinchonidine, kokusagine, orixine, and dictamnine are some example of quinoline alkaloids.

Quinoline existing in several natural compounds (Cinchona alkaloids) displaying the broad range of biological and pharmacological activity. Quinoline has been found to possess antibacterial, antifungal, antimalarial, anthelmintic, anticonvulsant, anti-inflammatory, analgesic and estrogenic activity. Quinoline is usually used for making dyes, hydroxyquinoline sulfate and niacin as well as a solvent for resins and terpenes synthesis.

##### Indole or Benzopyrole

Indole is an oldest and the best studied aromatic bicyclic organic compound (Figure 3B) firstly obtained by Adolf Baeyer [69]. The indole structure can be found in many organic a compound that’s why also known as “privileged structures” as they exhibit high binding affinity for many receptors [70]. Owing to the excessive structural diversity of indoles, they become an important structural component in many natural products showing biological activities auxin, reserpine, tryptophol, ellipticine, vincristine, flinderole etc. [71]. They exhibit extensive pharmacological properties such as antipsychotic, antihypertensive, antitumor [72], anti-microbial, anti-parasitic, antimalarial activity particularly against the

*Plasmodium falciparum*. Indole and its derivatives have always been studied in different research capacities such as pharmaceuticals, agrochemicals, pigments, fragrances, and material science.

##### Non-Heterocyclic Alkaloids

Non-heterocyclic alkaloids also termed as proto-alkaloids or biological amines and are rarely found in nature. These compounds contain a nitrogen atom which is not a part of any ring system (Figure 3C). Examples of these include phenylethylamine, tropolone, steroidal, ephedrine, colchicine, erythromycin, and taxol. Also in this review, some of the commonly used non-heterocyclic alkaloids are described.

#### 1.2.3. Phenolic Acids

**Figure 4:**
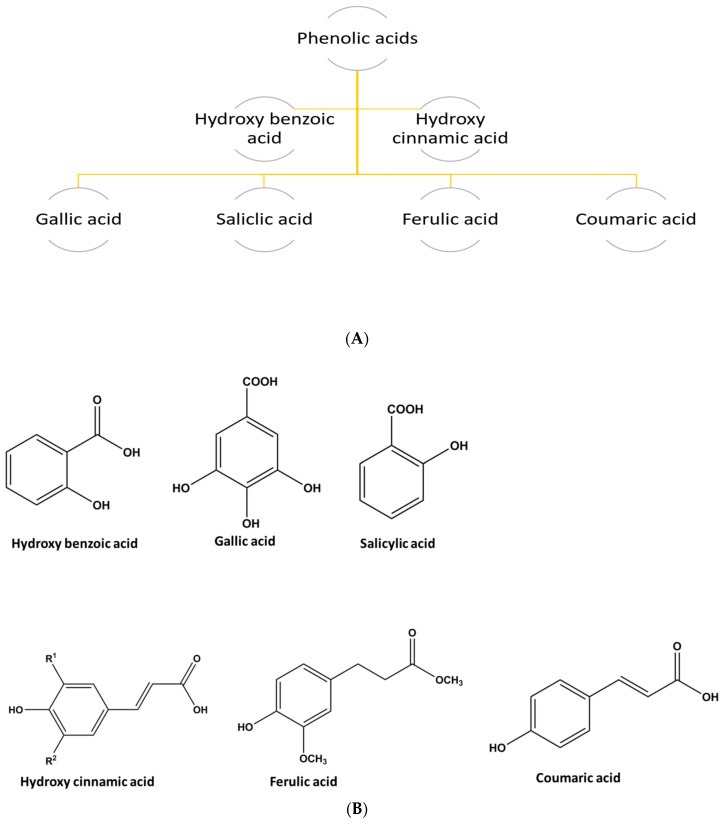
(**A**) Phenolic acids and their types (**B**) Common structure of some phenolic acids.

Phenolic acids are the plant secondary metabolites universally used to combat oxidative damage diseases (e.g., coronary heart disease and cancers) when ingested from fruits and vegetables [73]. Phenolic acids can be distributed into two groups: hydroxybenzoic acids and hydroxycinnamic acids (Figure 4A). The simplest phenolic acids found in nature are of group benzoic acids and cinnamic acids (Figure 4B) containing seven and nine carbon atoms in their structural conformation [74].

#### 1.2.4. Antibiotics

Antibiotics are the bioactive compounds with low molecular weight (MW 3000) produced by different organisms i.e., bacteria, fungi, algae, lichens, green plants, etc. via fermentation [75,76]. Antibiotics are generally defensive in function and often toxic to other species (e.g., penicillin, originally produced by bread mold, is toxic to numerous human pathogens). They generally act by inhibition of cell wall synthesis (group I), disruption of cell membranes (group II), interfering with protein synthesis (group III) interference with nucleic acid synthesis (group IV) and henceforth lyse the targeted cells involved [77].

Inorganic (e.g., Ag, Zn etc.) molecules may also have antibiotic properties. Antibiotics can be distributed into two groups: bactericidal i.e., those that kill bacteria and bacteriostatic means those that only impair bacterial growth. Furthermore, antibiotics are also classified based on their chemical structure and mechanism of action. It has been reported by several researchers that most of the antibiotics used target bacterial functions or growth. Antibiotics like ß-Lactams and glycopeptides target the bacterial cell wall or cell membrane is typically bactericidal in nature whereas aminoglycosides, tetracycline and macrolides target protein synthesis are bacteriostatic in nature [78]. Sulphonamides is a typical category of bacteriostatic antibiotics block the folic acid biosynthetic process in some microorganisms including bacteria required for nucleic acids synthesis, while humans lack this function and acquire folate through their diet [79]. Interestingly, antibiotics are further categorized based on their target specificity; for example, antibiotics will work differently for both Gram-negative and Gram-positive bacteria, respectively.

##### β-Lactams

**Figure 5:**
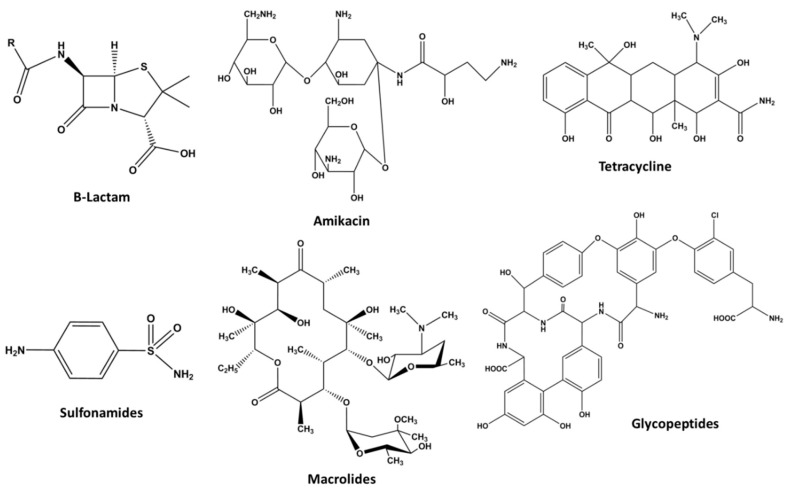
Common structure of some major antibiotic classes.

β-Lactams is a class of antibiotics having beta-lactam ring present altogether in their molecular structures (Figure 5) (e.g., penicillin G, ampicillin, cephalosporins, monobactams, etc.) [80] that kill targeted microorganism by modifying their essential cellular function that maintain cell wall (peptidoglycan) synthesis (creation/repair) or degradation in balance [81,82]. This class of antibiotics cause cell wall disruption resulted in the death of the targeted bacteria (pathogens). Further, they are species-specific to certain pathogenic bacteria (e.g., *Streptococcus*, *Meningococcus*, and *Diphtheria*) and do not harm other species (e.g., man).

β-Lactams are the extensively used group of all commercially available antibiotics [83]. Some pathogenic bacterial strains sometimes develop resistance to β-lactam antibiotics due to the production of β-lactamase, an enzyme that attacks the β-lactam ring [84,85]. To avoid resistance, β-lactamase inhibitors such as clavulanic acid, sulbactam, and tazobactam are often given with β-lactam antibiotics. Unfortunately, the available β-lactamase inhibitors do not inhibit all types of β-lactamases [86,87].

##### Aminoglycosides

Aminoglycosides structure comprises two or more amino sugars linked by glycosidic bonds to an aminocyclitol ring nucleus [88]. Aminoglycosides are broad-spectrum antibiotics used against severe infections caused by rapidly multiplying bacteria which are difficult to treat [89,90]. Furthermore, they possess high antimicrobial potential against aerobic and facultative Gram-negative bacteria like staphylococci and mycobacteria, etc. [91,92,93,94].

They are also frequently used to treat infections caused by *P. aeruginosa*, *Acinetobacter*, and *Enterobacter* that are described as resistant strains against multiple antibiotics [79]. These antibiotics bind to the 30S subunit of the ribosome and do not permit the binding of the 50S subunit to the initiation complex consequently inhibiting translation process required for their survival. They can differentiate between prokaryotic (70S) and eukaryotic (80S) ribosomes, and subsequently have a comparatively high therapeutic index. Other members of this group are gentamicin, tobramycin, kanamycin, streptomycin, and neomycin. However, spectinomycin is a bacteriostatic antibiotic chemically related to the aminoglycosides. These antibiotics have proved to have undesirable side-effects such as nephrotoxicity and ototoxicity; these have led to it being replaced in most applications by safer alternatives [88]. Further, the semisynthetic aminoglycosides include amikacin, dibekacin, and netilmicin exhibiting distinct toxicological profiles for strains that had developed resistance towards previously used aminoglycosides [90].

##### Tetracycline

The tetracyclines are generally bacteriostatic against atypical organisms such as chlamydiae, mycoplasmas, rickettsia, and protozoan parasites as well as Gram-positive and Gram-negative bacteria [91]. Tetracycline binds to the 30S subunit of the ribosome, inhibit the binding of aminoacyl-tRNA to the mRNA-ribosome complex reversibly, thereby inhibiting protein synthesis and bacterial cell growth. The antibiotics include tetracycline, doxycycline and minocycline. Tetracyclines have also shown immunosuppression, anti-inflammatory activity, inhibition of lipase and collagenase activity, wound healing and treatment of a variety of sexually transmitted diseases. In the case of pregnant women, tetracyclines should not be prescribed as the side effects subsequently appear in their babies like tooth discoloration [91].

##### Sulfonamides

Sulfonamides are the first modern anti-infective comparatively toxic bacteriostatic antibiotics. They specifically inhibit the conversion of *p*-aminobenzoic acid (PABA) to dihydropteroate and block microbial folate synthesis process [95]. Sulfonamides are generally used to treat infection caused by Gram-positive and Gram-negative bacteria, although it has poor activity against *P. aeruginosa* and strict anaerobes. Furthermore, sulfonamides effectively inhibit the growth of some types of protozoa and fungi. Due to toxic properties, these antibiotics should not be prescribed to pregnant women, neonates, and infants [96].

##### Macrolides

Macrolides are bacteriostatic agents comprises macro cyclic lactone ring to which one or more deoxy sugars are attached. They inhibit the translation process by binding reversibly to 50S ribosomal subunits of sensitive microorganisms. Erythromycin, clarithromycin, and azithromycin are the best known of the *macrolide* group of antibiotics. This class of antibiotics is active against Gram-positive strict anaerobic cocci while poor activity against enterococci, penicillin-resistant staphylococci and most Gram-negative bacteria with the notable exception of *Neisseria gonorrhoeae* [91,94].

Macrolides are mostly used for the treatment of staphylococcal and streptococcal infections as an alternative for patients who are allergic to β-lactam antibiotics.

##### Glycopeptides

Glycopeptides antibiotic are glycosylated cyclic or polycyclic non-ribosomal peptides initially obtained from soil bacteria and plants. They act primarily by disrupting the cell wall of susceptible microorganisms through inhibiting peptidoglycan synthesis. Glycopeptides are divided in to two groups: first-generation i.e., vancomycin, teicoplanin, ramoplanin and second generation include semi-synthetic antibiotics such as oritavancin, dalbavancin, and telavancin. Glycopeptides are extensively used antimicrobial agent against most Gram-positive organisms including multi-resistant *Staphylococci (MRSA)* although their activity tends to be limited towards different Gram-positive organisms [92]. Glycopeptides particularly vancomycin is considered as the last effective line of defense [97].

## 2. Food Processing Industries and Their By-Products

**Figure 6:**
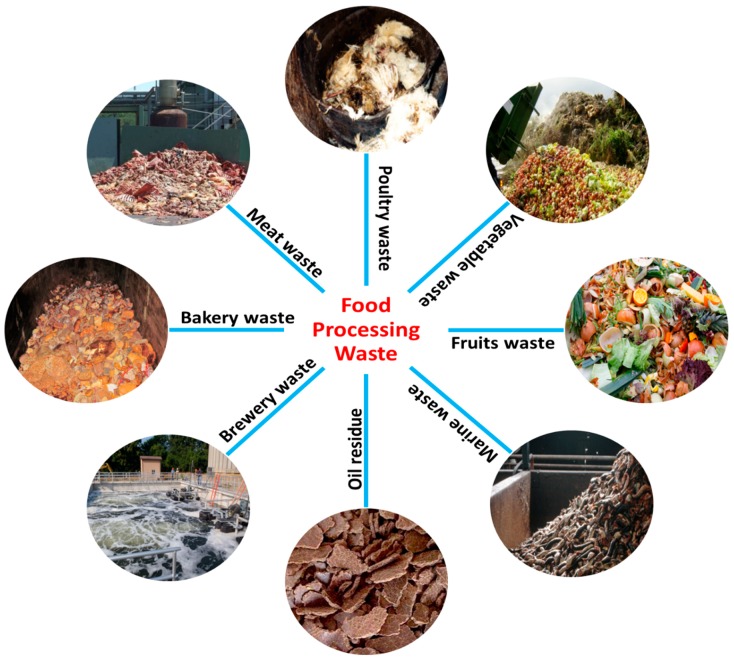
Food processing wastes from various food industries.

Food processing industries are the industries that used various methods as well as techniques to transform the raw materials into food or food into other forms for human consumption. There are so many food processing industries worldwide and Indian food handling industry is the world’s second largest producer of food or their relative products after China. Food processing industries include fruits and vegetables, dairy, meat, poultry, marine, brewery, and grain processing industries, which produced a huge amount of residue (waste) as by-products (Figure 6).

These food industries discard their waste in the environment, only some of them re-processed their waste and used as functional food ingredients. The Food and Agriculture Organization of the United Nations (FAO) evaluates that, every year, around 1.3 billion tons of food produced for human consumption in the world is vanished or wasted. This includes 45% of all fruit and vegetables, 35% of fish and seafood, 30% of cereals, 20% of dairy products and 20% of meat [98].

### 2.1. Fruit & Vegetable Processing Industries

There are numerous industries which are based on fruits as well as vegetables e.g., juice industries, pickle industries, oil industries etc. These industries processed the substrate for increases their shelf life by using canning, drying, freezing, and preparation of juices, jams, and jellies etc. The fruit and vegetable industries usually produce a huge amount of effluents as well as solid waste. The main solid waste constitutes organic materials, including discarded fruits vegetables, peel/skin, seeds, stones etc. whereas the effluents contain liquid waste of juice and wash waters.

In India, Asia’s largest vegetable, fruit and flower market i.e., Koyambedu market, Chennai spread over an area of 60 acres and produced approximately 80 tons of solid waste per day [99]. Subsequently, there is a major issue in regards to squander transfer, which can prompt issues with flies and rats around the preparing room, if not accurately managed. In most of the Asian countries there is shortage of feed for livestock e.g., in India a deficiency of 25, 159 and 117 million tons of concentrates, green forages and crop residues [100], China, have a shortage of 10, 30 and 20 million tonnes of protein feed, energy feed, and aquatic feed, respectively [101]. To overcome this problem, the fruit and vegetables waste has been used as an alternate source of livestock feed.

### 2.2. Meat & Poultry Industries

Meat and meat products form a vital section of the human diet because they are the good source of bioactive compounds [102] and give the vital nutrients that cannot be effectively acquired through vegetables and their determined items [103,104]. Thus, as the demand increased significantly, various industries are developed to fulfill the requirements. The impact of various livestock industries are increasing substantially in the GDP of a country which accounts for >40% of the total agricultural sector and >12% of GDP [105]. With the increase of meat and poultry industries, the productions of by-products are also increased due to non-utilization or low utilization of by-products.

Meat side-effects are created by butcher houses, meat processors, wholesalers, and rendering plant. Meat industries waste contains a high concentration of nitrogen, phosphorus, and grease depending on the type of waste. The animal blood, a kind of meat by product is an important edible by-product because it contains a high level of protein as well as iron [106]. In Asia, blood is used to make blood curd, blood cake and blood pudding [107]. Similarly, In Europe, animal blood is used in blood sausages, blood pudding, biscuits as well as bread. Animal blood has also been used in food as an emulsifier, stabilizer, clarifier, color additive, nutritional component etc. [108] and also used in pharmaceutical industries. Bah et al. [109] describes that the animal blood collected from various slaughterhouse has an emerging source for different bioactive compounds. So, with the improved or enhanced utilization of meat by-products can give a good alternative to reduce the huge amount of meat industries waste.

### 2.3. Dairy Industries

The dairy industries primarily comprise processing of raw milk into various foodstuffs like consumer milk, condensed milk, butter, cheese, dried milk (milk powder), yogurt and ice cream, using methods like chilling, pasteurization, and homogenization. As the demand for milk and their related products increases continually, the dairy industries also have grown rapidly in India [110]. Likewise, the waste generated from these industries caused serious environmental problems. Dairy industries produced waste especially in the form of water by different operations used during processing of milk [111]. These water effluents contain significant quantities of organic milk products, minerals, dissolved sugars, proteins, fats and possible residues of additives [112].

### 2.4. Marine Industries

Marine industries comprise seafood’s which prominently includes fish and shellfish. These are naturally practical and consumed by humans as food [113]. In fresh water fish production, India is the second biggest producer of fish around the world. Fish processing plants also produced a huge amount of waste and India alone generates greater than 2 million metric tons of by-products due to fish processing activities [114].

Similar to other food industries, fish processing setups produced waste in the form of solid like fish carcasses, viscera, skin, heads and liquid form like washing and cleaning water discharges, blood water from drained fish storage tanks, brine etc. [115]. Seafood’s and their by-products are an abundant source of nutraceutical and bioactive compounds. These can be extricated/secluded and added to a scope of sustenance’s, therefore updating handiness of the nourishment as far as human well-being [116].

### 2.5. Grain Processing Industries

India delivers in excess of 200 million tons of various sustenance grains each year. Add up to sustenance grains generation achieved 270.10 MT in FY16 (according to Ministry of Agriculture). Grain processing includes the handling of all types of grain, seed, granules and other bulk materials as well as vegetable oil, and comprises cleaning and grading, drying, seed processing, conveying and out loading, storage, vegetable oil processing, control-automation, aspiration, and filtration. The oil extraction process from the grain produced a huge amount of by products as waste (oil cakes) [117].

Similarly, biodegradable waste is created in each phase of grain handling, including the waste-water and air outflows treatment forms. Their administration causes some natural and money related issues [118]. By-products generated from the processing of cereals, pulses and other grains are promising sources of nutrients, including bioactive compounds (e.g., phytochemicals) which could be used for their favorable technological or beneficial nutraceutical properties [119].

### 2.6. Brewery Industries

The brewing industry is one of the leading consumers of water. The blending business utilizes various clump write activities in preparing crude materials for the last brew item. Simultaneously, vast amounts of water are utilized for the generation of the brew itself, and to wash, cleaning and sanitizing of different units after each cluster are finished [120]. Likewise, brewer spent grains, residual brewing yeast and trub called wet brewery wastes [121]. Most of the organic waste like spent malt and hops are directly used as animal feed and for soil improvement [122].

## 3. Fermentation Processes

Fermentation is one of the oldest approaches used for product transformation into value-added products through microorganisms. Mostly three types of fermentation processes are used such as solid state, sub-merged and liquid fermentation. Selections of the fermentation process are product specific. Solid state and sub-merged fermentation processes are used to obtained bioactive compounds of industrial interest from various substrates such as wastes. Both processes have been used for research as well as industrial level but some processes produced better yields than others did because the metabolism carried out by microorganisms is dissimilar in both processes [123].

### 3.1. Solid State Fermentation

Solid-state fermentation (SSF) is the fermentation procedure in which microorganisms develop on solid substrates in the lack of open liquid [124]. The main objective of SSF is to attain the maximum nutrient attention from the substrate for fermentation by using the microbes such as fungi or bacteria. SSF further classified on the basis of seed culture used for fermentation is pure or mixed. In pure culture SSF, specific strains are used whereas, in with the mixed culture, various microorganisms are used for fermentation.

On the basis of the nature of the solid phase, the SSF can be divided into two types. In the first type of SSF, the solid help to support as well as the nutrient source. These solid substrates are the by-products of grains and grain industries, cassava, potato, beans, and sugar beet pulp etc. and obtained from food various industries [125]. Whereas, in the second type of SSF the solid contributes support is soaked with a liquid medium (sugars, lipids, organic acids, etc.).

SSF potentials high volumetric productivity with increasing the concentration of products and reduced the effluent production [126]. However, a study performed using the organic waste for the production of enzymes by SSF stated the release of volatile organic compounds (VOC) such as CH_4_, N_2_O and NH_3_ [127]. Madhumithah et al. [128] carried out a study to produce protease from vegetable waste by SSF with *Aspergillus niger*. They used various vegetable wastes like potato, pumpkin, cauliflower, cabbage, and brinjal as a substrate for fermentation. They found maximum protein content i.e., 291.54 μg mL^−1^ from cauliflower among all the substrates. Similarly, Dhanasekaran et al. [129] obtained single cell protein using pineapple residues as a raw material through two different strains of yeasts, *Saccharomyces cerevisiae*, and *Candida tropicalis*. The biomass contents increase with the increase in the concentration of pineapple waste. The highest biomass and protein content was observed on the 7th and 3rd day of fermentation with both the yeasts.

SSF was also used for the extraction of lycopene using tomato waste as substrate by Jamal et al. [130]. Lycopene is an eminent carotenoid, producing the red colour of tomatoes and used as an antioxidant agent, colouring agent in the cosmetics, pharmaceutical as well as food industries. The effect of SSF by using fungus strain i.e., *Rhizopus oryzae* on the release of phenolic contents from rice bran was studied by Schmidt et al. [131]. A transformation in phenolic contents was observed, whereas ferulic acid giving the maximum enhancement during fermentation, initially from 33 mg/g in rice bran and finally 765 mg/g in the fermented bran.

### 3.2. Sub Merged/Liquid Fermentation

Submerged fermentation (SmF) is the type of fermentation in which the substrate is liquefied or put off in a water source. SmF is mostly used in industrial processes for high yield, low cost, and contamination. However, SmF has some disadvantages like physical space and energy or water requirements etc. [132]. Production of the enzyme by SmF has been used over past of century as compared to SSF because of some advantageous. This fermentation process is easier to plan by researchers because of the ease of process control and sterilization [133]. Pectinase, an enzyme production from fungi has been described by Favela-Tores et al. [134] using SmF. Pectinases are a gathering of related proteins engaged with the breakdown of pectin from an assortment of plants. Pectinases have various commercial as well as industrial importance.

Pectinase production also reported by Beg et al. [135]; Debing et al. [136] and Biz et al. [137] through fermentation processes using agro-wastes. Buyukkileci et al. [138] produced a high amount of enzyme i.e., exo-polygalacturonase from orange peel by using SmF with *Aspergillus sojae*. Corn husks were used as a substrate for the production of cellulose from *Bacillus cereus* by SmF [139]. SmF was performed for the amylase production using date wastes as a substrate with *Candida guilliermondii*. The process parameters such as incubation time, incubation temperature, initial pH, starch concentration, supplementary nitrogen source, nitrogen, and phosphorus concentrations that affect the production of α-amylase were also optimized [140].

Similarly, Budihal and Asgar [141] performed the production of cellulase enzyme by *Streptomyces* DSK29 under both types of bioprocesses such as submerged and solid state fermentation using agro wastes as substrates. Bioprocesses are the processes that convert the complex substrates into simple value-added products by various microorganisms. Likewise, Table 2 also describes the various utilization of food as well as agricultural base by-products using bioprocesses like fermentation.

## 4. Uses of Fermentation for the Production of Bioactive/Value Added Compounds

**Figure 7:**
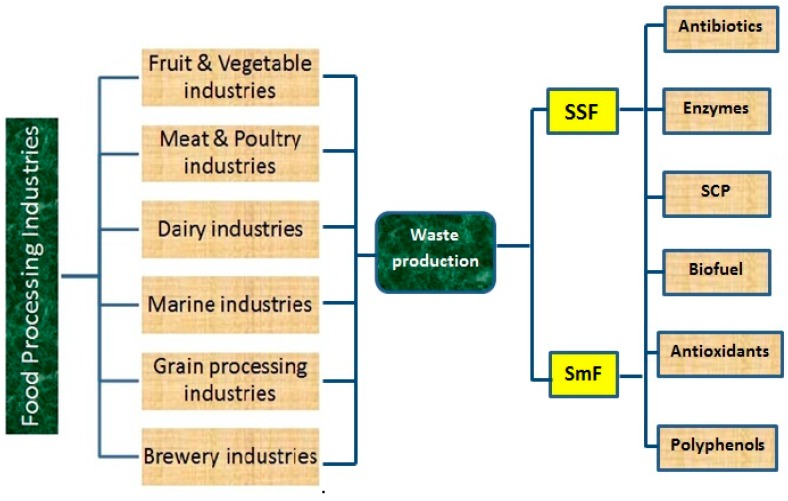
Production of bioactive compounds from food industries waste using fermentation.

SSF is a remarkable tool to elevate nutritional and functional values of the substrate to large extent [199,200]. Several types of solid substrates generated from agro waste have been used for solid state fermentation which is consist of high nutritive value in terms of proteins, fibers, and minerals, respectively [201]. Figure 7 shows the outline to production various bioactive compounds from food industries waste through fermentation. Owing to fact that these macro and micro molecules have tremendous value in human as well as animal diet, therefore to improve their digestibility and bioavailability solid-state fermentation is an effective approach [202,203]. Functional properties are the significant properties that define the pivotal phenomena of food, which are essentially used in food application [204,205]. Also, the functional properties of food are always correlated with intrinsic components such as proteins, starch, fats, respectively.

The functionality of these components is related to their physicochemical properties and molecule interactions as well as environmental conditions [206,207]. Several researchers explored the effect of solid state fermentation upon functional properties of agro-industrial waste and revealed the effectiveness of solid state fermented substrate in comparison with unfermented one [208,209]. Researchers also revealed that the solid state fermentation directly influences the protein structure and physicochemical properties, which in turn enhances the functional applications of the solid substrates originated from agro industrial wastes [210]. Among all functional properties, protein solubility is a significant property, which is directly influenced by the solid state fermentation. During this process, filamentous fungi act upon the proteins and structure of proteins tends to be open. After this, higher proteins unit are converted into smaller units, hence improve the solubility of the substrate into water system [211].

Another functional property is water and oil binding which are systematized by their configuration, structural behavior and the relations of proteins with each another and with added constituents, respectively [212,213]. Both water and oil binding capacities of the component are the principle process of protein-water and oil which take place in several food classifications [214]. Solid state fermentation directly affects the hydrophobic and hydrophilic domains of the components present in the solid substrates and significantly increase the water and oil binding properties. In addition, solid state fermentation has a tendency to open the protein structures which then attain the ability to absorb and hold bound, hydrodynamic, capillary and substantially entrapped water and oil against gravity.

Foaming property of components is the combination of both gas and liquid phase which mainly attained through the unfolding and absorption of the proteins at the air-water interface and forms the film all over the place of the air bubbles [199,206]. SSF significantly influences the cohesive nature of the proteins and due to this proteins rapidly diminish the surface tension at the air/water interface; they form large foam volume with large air cells. Also, decreased particle size of proteins after solid state fermentation improves the foaming properties and ionic strength of the protein. In this context, Sadh et al. [117] revealed significantly improved foaming properties of solid state fermented peanut press powder as compared to raw powder. Moreover, emulsifying activity and stability are important features as the functional property of food components that helps for emulsion formation and stabilization [206,207].

Solid state fermentation alters the physicochemical properties such as solubility, surface hydrophobicity, and molecular flexibility of solid substrates which ultimately affects the emulsion forming and stabilizing properties [199]. Moreover, emulsion stability is contingent on the degree of these interactions and solid state fermentation amends the solubility of proteins, ability to adsorb speedily at the interface, distribution of charged group and cohesive nature of the proteins, respectively [117,211]. Validation of the significantly improved emulsifying properties was agreed with findings of Sadh et al. [215] and Chawla et al. [199], respectively.

Owing to fact that proteins have noteworthy affinity to bind with the mineral ions electro-statisftically this interaction depends on the type of micronutrient and available sites on the proteins of the solid substrates [213]. Researchers unveiled improved functionality of the proteins present in solid substrates after solid state fermentation and due to these structural alterations bivalent metals tends to bind with available sites of the proteins. This complex formation with proteins mineral bioavailability significantly improved after SSF. Chawla et al. [199] and Sadh et al. [117] explained in vitro mineral bioavailability of the trace minerals after solid state fermentation of peanut press cake and black eyed seed powder, respectively. In their study, they compared the mineral bioavailability of these minerals with the inorganic form of the mineral salts and unveiled significantly increased cellular absorption and transportation across the Caco-2 cells, respectively. Also, ferritin synthesis was increased in solid state fermented samples as compared to an inorganic form of the salts.

Likewise, Chafle et al. [216] utilized vegetable and fruit waste for the generation of bioenergy in the form of biofuel. Fruit wastes constitute high reducing sugars, used for the production of bio-ethanol [217]. Whereas, the composition of vegetable wastes are high in cellulose, hemicelluloses, and lignin, which are used for the production of second-generation bioethanol [218,219,220].

Campo et al. (2006) performed a diluted acid hydrolysis process using fresh and processed vegetable wastes as substrate. They found highest ratios of only sugar in the liquid section obtained from dilute acid hydrolysis assays for tomato and red pepper residues. Similarly, Akin-Osanaiye et al. [221] investigated the production of ethanol from *Carica papaya* (pawpaw) agricultural wastes, by *Saccharomyces cerevisiae*. Their results revealed that the production of alcohol with fermentation of pawpaw fruit waste using baker’s yeast was 2.82–6.60% (*v*/*v*) in 72 h of fermentation. Potato peel waste was used for ligninolytic enzymes production like manganese peroxidase, laccase, lignin peroxidase and aryl alcohol oxidase using solid state fermentation by *Pleurotus ostreatus* [222].

Laccases are polyphenol oxidases enzymes, which have various impacts in bio-pulping, bio-bleaching, detoxification of environmental pollutants, pharmaceuticals, preparation of beverages etc. Dhillon et al. [223] obtained such enzymes from agricultural wastes like sugarcane bagasse, wheat bran, rice straw, and brewer’s spent grain. Similarly, diverse studies were performed using various agro-industries by-products i.e., coconut coir, wheat bran, sugarcane bagasse, and rice straw as a solid support for the production of laccase through different microorganisms such as *Pleurotus* sp., *Pleurotus ostreatus*, *Coriolus* sp., *Pyrenophora phaeocomes*, respectively [224,225,226]. Xylanases, type of enzymes which are used for hydrolysis of 1,4-α-d-xylosidic linkages in xylans, mainly obtained from the hemicellulose fraction of plant cell walls [227]. Among the different type of waste, wheat and rice bran are mostly used for the production of xylanase as reported in the various study [228,229,230,231,232,233,234].

The palm kernel press cake (PKC) was used in the different fermentation process for ethanol production because it contains monosaccharides. Cervero et al. [235] performed a study for ethanol production using PKC as a substrate through fermentation using *Saccharomyces cerevisiae*, resulting in ethanol i.e., 125 g kg^−1^ of PKC. Likewise, Hashem and Darwish, [236] also produced bioethanol via *Saccharomyces cerevisiae* using potato starch residue.

One of the most useful agricultural by-product i.e., rice bran was used for fermentation with *Aspergillus oryzae* and *Rhizopus oryzae* by Razak et al. [193]. Rice bran is attained through the milling of rice grain which has a great amount of protein approximately 12 to 15%, 11% fiber and 20% of its weight in oil [237,238]. Xia et al. [239] suggested that the protein present in rice bran contains well-balanced amino acid and also comprises a hypoallergenic protein which is required in baby food design. Razak et al. [193] evaluated organic acid, phenolics, antioxidants and anti-pigmentation activity etc. of fermented extracts of rice bran. Anti-pigmentation effect was found the maximum in rice bran extracts when fermented using *A. oryzae* such as 56.18% as associated with the additional extracts. Similarly, the anti-aging result was also presented with the maximum elastase inhibition activity with a value of 60.52%. A similar type of research also studied by Jamaluddin et al. [240] using rice bran as a substrate for fermentation with *Monascus purpureus* and *Aspergillus niger*, resulted in an augmentation in tyrosinase and elastase enzyme inhibition activity in fermented rice bran as compared to the non-fermented one.

Likewise, other antioxidants, phenolic activity, and organic acid were bio-transformed during rice bran fermentation [241,242]. Schmidt and Furlong [243], performed an experimental study to know the consequence of particle size and ammonium sulphate concentration of rice bran during fermentation with *Rhizopus oryzae* on the production of biomass, protein and phenolic contents. They concluded that the particle size of the substrate i.e., rice bran intensely influenced fermentation. Small particle size produced protein and phenolic content, whereas large particle size produced fungal biomass. Saykhedkar and Singhal [244], produced griseofulvin is a secondary metabolite using rice bran as a solid substrate for SSF with *Penicillium griseofulvum*.

Various studies on rice bran through fermentation process have been completed in previous years. Fermentation of rice bran from bacterial culture has been frequently used for the production of lactic acid [245,246,247]. Further rice bran has also been used for the production of biomass [248], antioxidants [131] and phenolic acids [131] enzymes like protease [149,182], cellulose [249] and amylase [250]. The protein concentrate of rice bran was enhanced using baker’s yeast at optimized condition by Chinma et al. [251].

Santa et al. [252] used *Beauveria bassiana* for SSF to obtain bio-pesticides from wastes such as potatoes, coffee husks to control the pests of banana, sugarcane, soybean, and coffee. Another fungus i.e., *Colletotrichum truncatum* has been used in SSF as mycoherbicide in contrast to the weed *Sesbania exaltata* by Pandey et al. [253].

Khiyami et al. [254] produced polyhydroxyalkanoates using *Bacillus* plastic composite support (PCS) biofilm and date palm syrup. Polyhydroxyalkanoates (PHAs) are a group of biopolymers that have wide structural variety and biodegradability. Thus, it refers to replacing synthetic plastics and making them future green materials. Similarly, date fruit and their related by-products are used in the fermentation process for the bioactive compounds. Various studies have been carried out using date and produced fermented products such as Lactic acid using *Lactobacillus* sp. KCP01 by Chauhan et al. [255]; Curdlan using *Rhizobium radiobacter* ATCC 6466 by Salah et al. [256]; Xanthan gum using *Xanthomonas campestris* by Moosavi-Nasab et al. [257]; Carotemoids using *Corynebacterium glutamicum* CECT690 and *Bacillus* spp. by Davati et al. [258] and Tavakkoli et al. [259]. Cheok et al. [260] presented a review of current trends of tropical fruit waste utilization. They described the recovery of health benefit bioactive compounds as cost free fruit wastes, so decrease the waste burden. They used tropical fruit wastes such as durian (*Durio zibethinus*), mangosteen (*Garcinia mangostana* L.), rambutan (*Nephelium lappaceum*), mango (*Mangifera indica* L.), jackfruit (*Artocarpushetero phyllus*), papaya (*Carica papaya*), passion fruit (*Passiflora edulis*), dragon fruit (*Hylocereus* spp.), and pineapple (*Ananas comosus*).

## 5. Concluding Interpretations and Future Approaching

Agro-industry especially the food industry produces a huge volume of wastes that obtained generally from processing setups. To find substitutes for recycling of these wastes is a major objective taken into account globally. The composition, quantity, and quality of wastes depend on the raw materials as well as the processing steps. Various types of food industries produces a different type of wastes like orange peel, wheat straw and bran, rice straw and bran, sugarcane bagasse, banana and potato peel, apple pomace, soybean waste, date syrup, oil press cakes, brewery waste, dairy waste, marine waste, food waste etc.

Appropriate applications i.e., fermentations used for biotransformation of these wastes into valuable products having low cost and high nutritive value. Undeniably, use of wastes not only excludes the dumping problems but also resolves the pollution-related problems. Therefore, extra governing endorsements, as well as principal funds, are essential to bring these value-added products in the commercial market. The valorization of agro-industrial by products to beneficial substances may not only provide future dimension to researchers but also decrease the existing environmental hazards.

## Figures and Tables

**Table 1:** Bioactive compounds in different food stuffs.

Food Sources	Bioactive Compounds	References
Apple	Epicatechin, catechins, chlorogenic acid, hydroxycinnamates, phloretin glycosides, quercitin glycosides, procyanidins, anthocyanins	[20,21,22]
Avocado	Epicatechin, catechin, gallic acid, chlorogenic acid, cyanidin 3-glucoside, homogentisic acid	[23]
Banana	Gallocatechin, anthocyanins, delphindin, cyaniding, catecholamine	[24,25,26]
Berries	Cyanidin, delphinidin, malvidin	[27]
Citrus	Hesperidin, naringin, eriocitrin, narirutin	[27,28]
Grapes	Cinnamic acid, coumaric acid, caffeic acid, ferulic acid, chlorogenic acid, neochlorogenic acid, *p*-hydroxybenzoic acid, protocatechuic acid, vanillic acid, gallic acid, proanthocyanidins, quercetin, resvaratrol, pullulan	[27,29,30,31]
Guava	Catechin, cyanidin 3-glucoside, galangin, gallic acid, homogentisic acid, kaempferol	[23]
Litchi	Cyanidin-3-glucosides, cyanidin-3-rutonoside, malvidin-3-glucoside, gallic acid, epicatechin-3-gallate	[32,33]
Mango	Gallic acid, ellagic acid, gallates, gallotannins, condensed tannins	[34,35]
Olives	Cyanidin, delphinidin, malvidin	[27]
Palm	Tocopherols, tocotrienols, sterols, and squalene, phenolic antioxidants	[36,37]
Pomegranate	Gallic acid, cyanidin-3,5-diglucoside, cyanidin-3-diglucoside, delphinidin-3,5-diglucoside	[38,39]
Carrot	Phenols, beta-carotene	[40]
Celery	Cyanidin, delphinidin, malvidin	[27]
Cucumber	Chlorophyll, pheophytin, phellandrene, caryophyllene	[41]
Onion	Quercetin, rutin	[27]
Tomato	Carotenoids	[42]
Parsley	Apigenin, luteolin, quercetin	[27]
Spinach	Apigenin; luteolin	[43]
Chenopodium	Apigenin; luteolin	[43]
Barley	β-Glucan	[44]
Rice	γ-Oryzanol, bran oil	[45,46]
Wheat	Phenolic acids, antioxidants	[47]
Beans	Daidzen, glycindin	[27]
Dark chocolate	Epicatechin	[48]
Green tea	(−)-epigallocatechin, (+)-gallocatechin, (−)epicatechin-3-*O*-gallate	[49]

**Table 2:** Various bioactive compounds produced from different microorganisms by fermentation using diverse food processing wastes.

Bioactive Compounds	Substrate	Microorganism	Fermentation Process	References
Single cell protein	Sweet potato, banana skin, orange peel, mango waste and pineapple peel; Dairy waste	*Saccharomyces* sp., *Saccharomyces cerevisiae*, *Candida tropicalis*, *Lactobacillus acidophilus*	Solid state fermentation; Liquid fermentation	[142,143,144,145]
Bioethanol	pineapple waste, banana waste	*Saccharomyces cerevisiae*,	Solid state fermentation	[146,147]
Indole-3-acetic acid	Cassava fibrous residue	*Bacillus subtilis*	Solid state fermentation	[148]
Protease production	Rice bran, Brewery waste (brewer’s spent grain, hottrub and residual brewer’s yeast); Soybean meal; Wheat bran, cotton seed meal and orange peel.	*Lactobacillus delbrueckii* ssp.; *Bacillus licheniformis*; *Aspergillus niger*	Liquid fermentation; Solid state fermentation	[149,150,151,152]
Lactic acid production	Dairy waste; rice bran, wheat bran, ragi bran, rice starch water, tea waste, sugar cane bagasse, groundnut and coconut oil cakes	*Lactobacillus* sp.; *R. oryzae* MTCC 8784	Fed batch fermentation	[153,154]
Ergosterol	Dairy waste (whey)	*Cryptococcus albidus* sp. *Aerius*	Liquid fermentation	[155]
Xanthan	Potato peel	*Xanthomonas citri*	Solid state fermentation	[156]
Protein	Orange peel	*Chaetomium* spp. *(KC-06)* and *Aspergillus niger*	Solid state fermentation	[157]
Phenolic content	Guava and pineapple waste; Peanut waste (peanut press cake); Rice bran; plum pomaces and brandy distillery wastes; pomegranate wastes	*Rhizopus oligosporus*: *Aspergillus awamori; Rhizopus oryzae*; *Aspergillus niger* and *Rhizopus oligosporus*; *Punica granatum*	Solid state fermentation	[117,158,159,160]
Antioxidants	Peanut waste (peanut press cake); apricot pomace; Apple pomace	*Aspergillus awamori*; *Aspergillus niger* (ATCC-6275) and *Rhizopus oligosporus* (ATCC-22959); *Phanerocheate chrysosporium*	Solid state fermentation	[117,161,162]
Neomycin	Apple pomace, cotton seed meal, soy bean powder and wheat bran	*Streptomyces fradiae NCIM 2418*	Solid state fermentation	[163]
Oxytetracycline	Groundnut shell, Sweet potato residues, Cassava peels, cocoyam peels	*Streptomyces Rimosus*, *S. vendagensis*, *S. speibonae*	Solid state fermentation	[164,165,166,167]
Rifamycin	Coconut oil cake, groundnut oil cake, ground nut shell and rice husk	*Amycolatopsis Mediterranean*	Solid state fermentation	[168]
Meroparamycin	Rice, wheat bran, quaker, bread, and ground corn	*Streptomyces* sp. strain MAR01	Solid state fermentation	[169]
Bleomycin	Date syrup	*Streptomyces mobaraensis ATCC*	Fermentation	[170]
Poly(3-Hyrdroxybutyric Acid)	Orange peel	*Bacillus subtilis*	Batch fermentation	[171]
Laccase	Peels of citrus fruits, soybean meal, tofu dreg, Brewer’s spent grain	*Rheinheimera* sp., *Lysinibacillus* sp., *Trametes versicolor*	Sub merged fermentation; Solid state fermentation	[172,173]
Bioherbicide	Soybean bran, bagasse and corn steep liquor	*Phoma* sp.	Solid state fermentation	[174]
Biosorbents	Apple pomace	*Aspergillus niger*	Solid state fermentation	[175]
Astaxanthin (pigment)	Wheat waste; olive pomace; bakery waste	*Yamadazyma guilliermondii, Yarrowia lipolytica*; *Xantophylomyces dendrorhous*, *Sporidiobolus salmonicolor*; *Monascus purpureus*	Solid state fermentation	[176,177,178]
Bioactive phenolic compounds	Wheat straw, Rice straw, Corn cob, Pea pod, Sugarcane baggase	*Aspergillus fumigatus*, *A. terreus*, *A. wentii, Penicillium citrinum*, *P. granulatum, P. expansum*	Solid state fermentation	[179]
Fibrinolytic enzyme	Banana peel, black gram husk, paddy straw, rice bran, and wheat bran	*Bacillus halodurans* IND18	Solid state fermentation	[180]
Pectin lyase	corn steep liquor and orange peel	*Aspergillus brasiliensis*	Sub merged fermentation	[181]
Citric acid	Apple pomace, brewer’s spent grain, citrus waste, sphagnum peat moss; peanut shell	*Aspergillus niger* NRRL 2001; *Aspergillus ornatus* and *Alternaria alternata*	Solid state fermentation	[182,183]
Fumaric acid	Apple pomace; pulp and paper solid waste	*Rhizopus oryzae 1526*	Solid state fermentation; Sub merged fermentation	[184,185]
Biosurfactant	Potato peels, orange peels, banana peels, and bagasse	*Bacillus subtilis* ANR 88	Solid state fermentation	[186]
Wine (antioxidant rich)	Potato, pumpkin and carrot peels	*Saccharomyces cerevisiae (NCIM 3206)*	Liquid fermentation	[187]
Cellulase	Wheat bran, Rice bran, Corn husks	*Trichoderma viride*, *Bacillus cereus*	Sub merged fermentation	[188,189]
Lycopene	Tomato waste	*Aspergillus niger*	Solid state fermentation	[130]
Polygalactouronase	Wheat bran, Coffee pulp	*Aspergillus niger*	Solid state fermentation	[190]
Vanillic acid and vanillin	Pineapple canary waste	*A. niger I-1472* and *Pycnoporus cinnabarinus MUCL 39533*	Liquid fermentation	[191]
Proanthocyanidins, anthocynidins, phenolic acids, vitamin E and oryzanol	Rice bran	-	-	[192]
Ferulic, p-coumaric, sinapic and syringic	Rice bran	*Aspergillus oryzae* and *Rhizopus oryzae*	Solid state fermentation	[193]
Lipase	Castor bean waste; *Jatropha curcas* seed cake; Sugarcane bagasse, sunflower seed and olive oil	*Penicillium simplicissimum*; *Pseudomonas aeruginosa*; *Burk holderiacenocepacia*; *Thermomucorindicaeseudaticae*	Solid state fermentation	[194,195,196,197]
Nisin	Date by product	*Lactococcus lactis*	Solid state fermentation	[198]
